# Acceptance of Diversity as a Building Block of Social Cohesion: Individual and Structural Determinants

**DOI:** 10.3389/fpsyg.2021.612224

**Published:** 2021-03-04

**Authors:** Regina Arant, Mandi Larsen, Klaus Boehnke

**Affiliations:** ^1^Department of Psychology and Methods, Jacobs University Bremen, Bremen, Germany; ^2^Bremen International Graduate School of Social Sciences (BIGSSS), Jacobs University Bremen, Bremen, Germany; ^3^Center for Sociocultural Research, National Research University – Higher School of Economics, Moscow, Russia

**Keywords:** acceptance of diversity, intergroup anxiety, empathy, political orientation, social cohesion, group-level outcome, multilevel analysis

## Abstract

High levels of social cohesion have been shown to be beneficial both for social entities and for their residents. It is therefore not surprising that scholars from several disciplines investigate which factors contribute to or hamper social cohesion at various societal levels. In recent years, the question of how individuals deal with the increasing diversity of their neighborhoods and society as a whole has become of particular interest when examining cohesion. The present study takes this a step further by combining sociological and psychological approaches in investigating whether the *group-level acceptance of diversity*, a core feature of cohesive societies, is related to prevailing mentalities of individuals once the social structure of a community is accounted for. We hypothesize that after controlling for individual sociodemographic and for structural variables, three individual characteristics play an important role for the level of acceptance of diversity in a given entity. We propose that individual intergroup anxiety (IGA) acts as a motor of the rejection of diversity whereas individual empathy should act as a safeguard. Furthermore, we propose that right-leaning political orientation (PO) has a negative influence on the acceptance of diversity. This study is based on a large, representative sample of the German general population (*N_1_* = 2,869). To draw comparisons among different social entities, the sample was divided by federal states (*N_2_* = 16). Data were analyzed by using a two-step approach for analyzing group-level outcomes in multilevel models. The analyses confirmed our hypothesis that intergroup anxiety at the individual level hampers the acceptance of diversity in a given sociopolitical entity. Furthermore, we found that intergroup anxiety is impacted by the economic situation in a federal state (measured per capita gross domestic product), as economic weakness intensified the fear of others. Surprisingly, neither empathy nor political orientation played a role for the acceptance of diversity. Implications for future research on social cohesion as well as for the work of policy makers are discussed.

## Introduction

Growing diversity is a fact in many societies today. In public discourse, migration is usually identified as the main driver for diversification. Continuous global migration movements since the end of the Cold War, and even more so since the beginning of the 21st century (Tessmer, 1994; Veser, 2015), have led to increasing ethnic diversity accompanied by greater religious and linguistic variety in what have been labeled Western, educated, industrial, rich, and democratic (WEIRD) societies (Henrich et al., 2010). At the same time, public discourse about the significance of increasing diversity for social processes is often not only heated, but also based on conjecture. What has drawn much attention in past years is the investigation of the possible influence of ethnic diversity on social cohesion (Babacan and Herrmann, 2013; Sturgis et al., 2014). The reason why social cohesion has attracted the interest of a growing number of societies around the world (European Commission, 2013; OECD, 2014) is the assumption that high levels of cohesion have beneficial outcomes both for the individual as well as for society as a whole. Indeed, research consistently demonstrates that residents of cohesive communities show higher subjective well-being (Delhey and Dragolov, 2016; Dragolov et al., 2016), better health (Arant et al., 2016, 2017), and a more positive emotional development (Reeve et al., 2016). In other words, individuals are happier, healthier, and emotionally more stable when they live in cohesive places than in communities with weaker social cohesion.

It is therefore not surprising that the number of studies investigating the impact of diversity on social cohesion, particularly from the fields of political science and sociology, has increased significantly in recent years. The starting point for much of this research is the seminal work of Putnam (2007) on diversity in modern communities (e.g., Dinesen and Sønderskov, 2012; Neymotin, 2014). Based on the results of a study with a large general United States population sample, he argues that ethnic diversity weakens social capital and thereby social cohesion. At first sight, this conclusion seems to resonate with the principles of conflict theory (Blumer, 1958), which assumes that diversity causes perceptions of threat and in turn lowers trust in outgroups. However, Putnam goes a step further and extends this mechanism to neighborhood and even ingroup trust. He argues that “[…] people living in ethnically diverse settings appear to ‘hunker down’ – that is, to pull in like a turtle” (Putnam, 2007; p. 149). Although there is a substantial number of studies supporting the presumed negative link between diversity and social cohesion, to the extent that it appears to become an “empirical regularity” (van der Meer and Tolsma, 2014, p. 471), there are also studies contradicting this association (see Portes and Vickstrom 2011; Schaeffer, 2013b). In their review, van der Meer and Tolsma (2014) indeed find a positive link between ethnic heterogeneity and trust between members of different ethnic groups. Furthermore, they find that apart from trust, minimal contact between highly diverse neighbors does not affect other aspects of social cohesion, such as helpfulness or voluntary commitment.

In examining the reasons for these inconclusive results, many studies bear at least one of three major issues: an oversimplified operationalization of social cohesion, a focus on objective characteristics of diversity (e.g., the degree of diversity in a certain community) instead of investigating individual (subjective) perceptions of it, and finally a narrow understanding of diversity as referring to ethnic diversity only. We discuss these points in the following, as they constitute the gaps in the literature addressed by the present paper, and then go on to mention individual characteristics that have proven to be important for understanding acceptance of diversity, namely people’s intergroup anxiety (IGA), their level of empathy, and their political orientation (PO). However, the literature that we discuss investigates this link on the individual level only. How such individual attributes impact the acceptance of diversity on the societal level is still largely unclear. To understand the relationship between emotions, attitudes, and behaviors of the individual and its social environment is, however, of utmost importance when one wants to understand processes of change on the macro level.

The first issue identified above refers to the operationalization of social cohesion. Putnam and many others use trust in various groups as a proxy for social capital and cohesion (Putnam, 2007; Uslaner, 2012; Larsen, 2014; Helbling et al., 2015; Laurence et al., 2019). Although there is still some conceptual disagreement about the definition of social cohesion, scholars across disciplines agree that it is not a unidimensional construct, but consists of a variety of aspects (for a review, see Schiefer and van der Noll, 2017). Using trust as the single indicator for social cohesion therefore misses out on the concept’s multidimensionality (Laurence, 2011).

One of the more influential attempts to address this issue has been the *Bertelsmann Social Cohesion Radar* (SCR; Dragolov et al., 2016). The SCR conceptualizes social cohesion as a multidimensional construct, allowing for a detailed and comprehensive measure. It encompasses three domains with three separate dimensions each, and assesses the level of social cohesion in a given social entity *via* a formative index, adding scores for the nine dimensions. Social relations (Domain 1) encompass the quality of social networks, the trust people have in others as well as the *acceptance of diversity*, which is at the core of the current research. Connectedness (Domain 2) is measured *via* the degree of identification with the social entity (nation, federal state, region, or neighborhood), trust in institutions, and the subjective experience of justice. Finally, Domain 3, the focus on the common good, entails solidarity and the degree to which people are willing to help others, the willingness to abide by basic social rules, and the degree to which people participate in civic life and political processes. In contrast to research focusing on trust as a proxy for cohesion, studies performed with this multidimensional index did not find that diversity by itself has a negative impact on cohesion. On the contrary, the relationship between diversity and cohesion is either insignificant (Dragolov et al., 2013; Arant et al., 2017) or even positive (Arant et al., 2016; Dragolov et al., 2014).

Instead of focusing on the objective level of diversity as a factor influencing social cohesion – as it is broad practice in the literature – the SCR incorporates acceptance of diversity as a dimension of social cohesion. Thereby, the individual’s handling of societal heterogeneity as “negotiated difference” (Dukes and Musterd, 2012, p. 1985) becomes a central element for the assessment of the quality of social cohesion. This conceptualization is not overly new, but addresses the second point of criticism mentioned above. In positing acceptance of diversity as a component of social cohesion, the SCR is in line with a broad range of scholars and institutions (Cassiers and Kesteloot, 2012; Novy et al., 2012; Office of Ethnic Communities, 2016).

Most research that focuses on the impact diversity can have on the individual and which individual preconditions influence this relationship stems from social psychology. Those studies are based on variations of conflict theory (e.g., Blumer, 1958) as well as integrated threat theory (Stephan et al., 2000). Stephan and Stephan (1985) argued early on that anxiety about a foreign culture leads to an increased orientation toward ingroup norms, which may result in discrimination in the case of perceived threat. Although the work of Putnam (2007) is based on the very same theoretical assumptions, he ignores the effects of intergroup contact and perceived intergroup threat. Recent studies therefore are trying to disentangle the link between levels of diversity and social cohesion by specifically taking the *acceptance* of diversity into consideration.

Although the overall association between increasing levels of diversity and decreasing social cohesion seems to be rather robust, various studies found individual characteristics to affect this relationship. Laurence et al. (2019) show, for example, that levels of diversity only reduce neighbor-trust among individuals who already viewed outgroups as threatening. In a study of 50 neighborhoods from 16 West German cities, Stolle et al. (2013) could not confirm the negative effect of neighborhood diversity mostly reported for the United States and for certain other European settings. Furthermore, the authors found that interethnic contact moderated the effect of increasing diversity. The first study that put Putnam’s hypotheses to a full test (Schmid et al., 2014) found that although objective measures of diversity correlated with lower outgroup and neighborhood trust in a White British sample, it was not associated with ingroup trust or outgroup attitudes. At the same time, individuals in neighborhoods with greater diversity reported increased interethnic contact, which correlated with reduced perceptions of intergroup threat. In other words, the direct negative effects of diversity were canceled out by indirect effects of intergroup contact. Taken together, recent psychological research provides convincing evidence that instead of the actual level of diversity in a given entity, rather the acceptance of other ethnic groups, as well as intergroup contact, are relevant for the level of social cohesion.

The third point of critcism addresses the fact that in public discourse there is often a narrow understanding of diversity. The work by Putnam and others on factors affecting social cohesion mirrors the public debate as it predominantly focuses on ethnic diversity as well as linguistic and religious diversification (c.f. Schaeffer, 2013a; Schmid et al., 2014). However, this view is limited considering that the growing number of migrants is not the only source of increasing diversity – and perceived threat – in contemporary societies. Religious diversification, for example, not only originates from the growing proportion of non-Christian beliefs, but also from a continuous secularization process, as for example in Germany. While in 1950 about 96% of the West and East German population indicated a Catholic or Protestant belief (Federal Anti-Discrimination Agency, 2016), meanwhile atheists and people not affiliated to a religious denomination constitute the largest group (39%), larger than Roman Catholics (27%), Protestants (25%; mostly Lutherans), and Muslims (5%; FOWID, 2020). Furthermore, various societal groups, such as persons with disabilities, women, and the LGBTIQ+-community, actively demand equal rights and treatment, by which they gain visibility.

Sociology calls this development the individualization and pluralization of life forms (Huinink and Wagner, 1998). Demographic change, gender mainstreaming, as well as higher life expectancies and falling birth rates add to the complexity of societal diversification. While men increasingly take on new roles (Dosch, 2016), voluntary childlessness can be observed in more and more women, particularly among the highly educated (Boehnke, 2010). On top of that, social classes apparently are losing their strong boundaries and their significance for individuals (Wiesendahl, 2017), while the inequality of income and wealth has increased considerably within the past 20 years (Grabka and Westermeier, 2014), causing growing societal imbalances and a variety of living conditions across individuals. Taken together, in the majority of Western societies, diversity increases not only because of migration, but also due to various drastic societal changes, which must be taken into account when investigating the link between diversity and social cohesion.

In order to address these gaps in the literature, we intend to investigate whether the acceptance of diversity (according to a multidimensional measure) in a given entity is primarily a consequence of the social structure or a matter of mentalities prevailing among residents of that entity. Intergroup anxiety and individual empathy will receive special attention, as will political orientation. As mentioned above, this paper is interested in the impact of individual level characteristics on the group-level acceptance of diversity. However, research usually focuses on one level only. Particularly, the link between individual-level intergroup anxiety and acceptance of diversity is rather established. Based on the aforementioned work by Stephan and Stephan (1985), studies continuously show that intergroup anxiety is associated with elevated levels of prejudice (Islam and Hewstone, 1993; Britt et al., 1996; Stephan et al., 1999; Stephan et al., 2000; Laurence et al., 2019), which in turn leads to an orientation toward one’s own group and may result in discrimination of outgroups in case of perceived threat.

While intergroup anxiety can be seen as a motor of the rejection of others, empathy has been found to function as a safeguard against the rejection of otherness. Being considered a relatively stable personality trait related to agreeableness (Graziano and Eisenberg, 1997), empathy seems to be linked to tolerance (Vogt, 1997; Witenberg, 2007), and pro-social behavior (Eisenberg et al., 1995; Hoffman, 2000). It is, thus, not surprising that studies consistently find that empathy has positive effects on attitudes and behavior toward others (Finlay and Stephan, 2000; Cowan and Khatchadourian, 2003), including various societal groups, such as ethnic minorities (Dovidio et al., 2004; Butrus and Witenberg, 2013), non-native speakers (Madera et al., 2011), convicted murderers (Batson et al., 1997a), people with AIDS (Batson et al., 1997b), the elderly (Galinsky and Ku, 2004), drug addicts (Batson et al., 2002), and the homeless (Batson et al., 1997b).

Furthermore, there is research on the relationship of people’s political orientation and outgroup attitudes and behavior. Right-wing orientations have been found to correlate with a greater need for stability and security, as well as the wish to maintain the status quo, particularly in Western Europe (Jost et al., 2003; Thorisdottir et al., 2007). Therefore, compared to individuals with more moderate political orientations, people who allocate themselves on the right-end of the political spectrum tend to avoid cultural change, endorse traditionalism and conformity, and justify inequalities (e.g., Jost, 2006; Wetherell et al., 2013), which has also been found to lead to negative feelings toward minority groups and outgroup derogation (Van Prooijen et al., 2015). In their study, Verkuyten et al. (2016) investigated the relationship between political orientation and the feeling of common national belonging which represents an appreciation of diversity and societal inclusion. The authors found that individuals with a stronger right-wing orientation showed lower endorsement for a common national belonging, irrespective of whether they were native to the country or if they had migrated to the Netherlands. Similarly Zeigler-Hill et al. (2020) showed that there is an interrelation between individuals’ social worldviews, political orientation, perceived threat, and attitudes toward peace with Palestine. Right-wing authoritarianism seems to play a moderating role there. In a three-country comparison of Poland, the Czech Republic, and Germany, Rippl et al. (2007) show that perceived threat is related positively to a self-reported political stance on the right in Germany. We therefore assume a negative relationship between right-wing orientations and the acceptance of diversity in Germany.

In conclusion, the current research proposes that the acceptance of diversity in a community is central for the level of social cohesion in a given entity. The pressing question that arises from this observation is which factors contribute to acceptance (or rejection) of diversity in a given social entity. Psychological research in this area usually focuses on individual preconditions that affect individual-level acceptance of otherness, but does not address their impact on community-level acceptance of diversity. The present study aims at overcoming this gap. While taking a clearly social psychological stance, its goal is to investigate if the acceptance of diversity in a social entity is related to prevailing mentalities of individuals once the social structure of a community is accounted for. By combining macro and micro level approaches, this paper not only adds to social psychological literature on the acceptance of diversity, but also addresses pressing sociological questions about the influence of citizens’ emotions, attitudes, and behaviors on life in diverse societies. Thereby, the paper aims at making a contribution relevant also to policy makers and actors in civil society. Unlike most studies in psychology, the reported research relies on representative survey data from the 16 federal states of Germany, thereby offering higher levels of generalizability of its findings than the usual convenience sample approach dominating much of the work on stereotypes and prejudice (Hewstone, 2015). Although the reviewed literature above only refers to research on the individual level, we transfer the findings to investigate the relationship between individual-level characteristics and group-level acceptance of diversity to test the following hypotheses, thereby applying the isomorphism assumption, nicely described in an essay of Galtung (1990) on the relationship between biography and history.

*Hypothesis* 1: Individual intergroup anxiety will be negatively related to acceptance of diversity at the level of the German federal states, even after controlling for individual sociodemographics and structural characteristics of the federal states.
*Hypothesis* 2: Individual empathy will be positively related to acceptance of diversity at the level of the German federal states, even after controlling for individual sociodemographics and structural characteristics of the federal states.
*Hypothesis* 3: Right-leaning political orientation will be negatively related to acceptance of diversity at the level of the German federal states, even after controlling for individual sociodemographics and structural characteristics of the federal states.

## Materials and Methods

### The Data Set

The data used for our analyses stem from the research project *Cohesion in Diversity* commissioned by the Robert Bosch Stiftung (Foundation) to examine acceptance of diversity across the German federal states (Arant et al., 2019). Data were collected *via* standardized telephone survey interviews with German-speaking persons age 16 and above (infas Institute for Applied Social Sciences, 2018). A dual frame approach was applied, whereby the numbers were selected in a ratio of 55:45 landline to mobile numbers, allowing for nearly full coverage of the population. Telephone numbers were randomly selected from two sampling frames of all possible landlines and all possible mobile phone numbers in Germany. These are known as the ADM Sampling System for Landlines and the ADM Sampling System for Mobiles, which have been created by a group of market research agencies for the purpose of representative surveys (ADM, 2018). To foster random sampling within a household in calls to landlines, participants were selected by asking to speak to the person in the household who had most recently had their birthday. In calls to mobiles, the primary user of the mobile was asked to participate.

The goal of the research project *Cohesion in Diversity* was to carry out approximately 3,000 interviews across Germany [population 83.0 million in 2018 (Statista, 2020a)], but also to calculate federal state scores. In order to accomplish this, it was necessary to use a disproportionately drawn sample to carry out a sufficiently large number of interviews in each federal state. This meant that in the small federal states (e.g., Bremen), more interviews were performed (e.g., *n* = 79) than would have been the case with a proportional approach (e.g., *n* = 25). A total of 2,937 viable interviews were conducted from May to July of 2018, which lasted an average of 34 min each. For the purpose of our analysis, listwise deletion was applied to the individual-level variables, leaving an individual level sample of *N_1_* = 2,869 participants. Accordingly, the working sample size of federal states (*N_2_* = 16) ranged from 73 individuals in Saarland [population 990,509 in 2018 (Statista, 2020b)] to 449 individuals in Bavaria [population 13.1 million in 2018 (Statista, 2020c)].

In order to ensure a representative sample at both the national and federal state level, weighting was applied in the analysis which took into account: (1) the probability of being selected into the sample according to the dual frame approach and the disproportionate sampling; (2) scaling so that the sum of the weights is equal to the number of realized interviews (Asparouhov, 2006); and (3) calibration according to the relevant benchmark values from the 2016 German microcensus (Federal Statistical Office and Statistical Offices of the Länder, 2016). At the national level, benchmark values included education and household size, while at the federal state level this included sex, age group, and community size.

### Individual-Level Predictors

#### Intergroup Anxiety

The level of intergroup anxiety was measured using a modified version of an instrument suggested by Stephan and Stephan (2000). Using a collection of eight items, participants were asked to indicate the degree to which they experienced a set of affective states (e.g., “worried,” “stressed”) when thinking of increasing diversity in Germany. The response scale ranged from 0 (“I do not have this feeling at all”) to 10 (“I strongly feel this way”). Positive affective states (e.g., “relaxed,” “satisfied”) were reverse-coded, and the eight items (*α* = 0.79) were then averaged, so that higher scores indicate greater intergroup anxiety (*M* = 4.29, *SD* = 1.61). Descriptive statistics of this and all subsequent measures can be found in Table 1.

**Table 1:** Descriptive information on variables used.

	Mean	SD	*Min*	*Max*
***Level 2: Federal state (N_2_ = 16)***				
ln(per capita GDP)	10.44	0.22	10.06	10.98
Acceptance of diversity	67.29	3.76	61.49	72.30
***Level 1: Individual (N_1_ = 2,869)***				
Intergroup anxiety	4.29	1.61	0	10
Empathy	4.15	0.66	1	5
Political orientation	4.41	2.11	0	10
Age (years)	51.34	17.81	16	97
Female	0.51	0.01	0	1
Marital status: married or cohabitating	0.63	0.01	0	1
Marital status: divorced, separated or widowed	0.18	0.01	0	1
Marital status: single	0.21	0.01	0	1
Education: University degree	0.34	0.01	0	1
Education: vocational training	0.51	0.01	0	1
Education: no completed secondary education	0.13	0.01	0	1
Employed	0.61	0.01	0	1
Migration background	0.14	0.01	0	1

SD, standard deviation

**Table 2:** Example indicators used for measuring the seven dimensions of acceptance of diversity.

Dimension of cohesion	Example indicator
Age	I do not get along very well with people who are clearly younger or older than me.
Disability	I find many demands from disabled people to be exaggerated.
Gender	I am against quotas for women.
Sexual orientation/gender identity	Transgender people should stay to themselves.
Cultural otherness/ethnicity	If I had a choice, I would rather have nothing to do with people from other countries.
Religion	Islam also fits into the Western world (−).
Economic disadvantage	We take too much care of people who are failures in our society.

A complete list of the 23 indicators can be found in Table 18 of Arant et al. (2019).

#### Empathy

Modified versions of two subscales of the Interpersonal Reactivity Index (Davis, 1983) were used to measure the level of empathy, namely aspects related to empathetic concern (feelings of concern toward unfortunate others) and perspective taking (the tendency to adopt the psychological viewpoint of others). Each subscale was assessed with three items, such as “I often feel touched by the things happening to the people around me” for empathic concern, and “Before I judge a person, I try to imagine how I would feel in their place” for perspective taking, using a Likert scale ranging from 1 (“Does not describe me well”) to 5 (“Describes me very well”). Principal component analysis with the six items indicated a one-factor solution, such that all six items (*α* = 0.75) measure one overall construct of empathy. Thus, all six items were then averaged to come up with a single score for empathy (*M* = 4.15, *SD* = 0.66), with higher scores indicating greater levels of empathy.

#### Political Orientation

Political orientation was measured by asking participants to locate themselves on a spectrum of political beliefs from 0 (“Left”) to 10 (“Right”; *M* = 4.41, *SD* = 2.11), so that higher scores indicate a more right-leaning political orientation. The format of the question is identical to the one used in the European Social Survey, Wave 9 (European Social Survey, 2018).

### Group-Level Dependent Variable

Our analyses use the Bosch Diversity Barometer (BDB) score calculated by the *Cohesion in Diversity* project (Arant et al., 2019) as our measure of acceptance of diversity at the federal state level. The BDB is unique in comparison to other measures of acceptance of diversity in that it incorporates seven key dimensions of social diversity: age, disability, gender, sexual orientation and gender identity, ethnicity, religion, and economic disadvantage. We describe here the calculation of the index by Arant et al. (2019) in order to provide sufficient background on its contents.

The BDB was calculated using the data as described in “The Data Set.” The first step involved exploratory factor analysis and internal consistency checks of all survey items related to the proposed seven dimensions of acceptance of diversity. The items included statements such as “Islam also fits into the Western world.” And “If I had a choice, I would rather have nothing to do with people from other countries.” Participants were asked for their agreement on a scale from 1 (“Do not agree at all”) to 4 (“Fully agree”). In order to simplify further analyses and allow for easier interpretation of the BDB by the general public, all items were transformed to a 100-point scale. For an item to be selected as a measure of a particular dimension, it had to meet an absolute factor loading of 0.40 or greater. This resulted in the selection of a total of 23 items, with three to four items per dimension^1^ and a Cronbach’s alpha indicating sufficient quality of each of the constructed dimensions of acceptance of diversity.^2^ The scores of the selected items for each dimension were then averaged across all individuals living in a federal state to create the overall score for each dimension at the federal state level, where 0 indicates full rejection of that aspect of diversity and 100 indicates full acceptance.

Arant et al. (2019) further tested their reflective measurement model by conducting an exploratory factor analysis to investigate whether each dimension adequately reflects the acceptance of diversity as a superordinate construct. All seven dimensions surpassed the factor loading threshold of 0.40, and were therefore retained as part of the BDB (*α* = 0.70). The final step involved then averaging the dimension score into one overall score from 0 to 100 (*M* = 67.29, *SD* = 3.76) for each federal state, where higher scores indicate greater levels of acceptance of diversity. Results of a skewness and kurtosis test indicate a normal distribution (*χ*^2^ = 1.13, *p* = 0.13).

### Individual-Level Covariates

Sociodemographic characteristics of participants were controlled for in the models. These included age (*M* = 51.34, *SD* = 17.81) and being female (51.2%). Likewise, marital status was included, with married and cohabiting respondents (62.9%) forming the reference group in comparison to the separated, widowed, and divorced (17.7%), and to singles (21.2%). The completed post-secondary education of participants was categorized into three groups, with those having a degree from a university or university of applied science (33.9%) serving as the reference group in comparison to those with vocational training (50.7%) and those who have not (yet) completed post-secondary education (13.5%). Participants were categorized as being employed if they indicated any level of current part- or full-time paid work (60.9%). Participants were also categorized as having a migration background (14.2%) if they themselves or at least one of their parents was not born in Germany.

### Group-Level Covariate

Several structural variables were initially selected for our analysis based on their significant partial correlation with federal state scores on the BDB after controlling for per capita gross domestic product (GDP), according to initial results by Arant et al. (2017): the unemployment rate for those aged 55–64 years old, income inequality as measured by the Gini-index, and access to high speed internet (> 50 Mbps). However, given that all of these structural variables were highly correlated with one another (ranging from *r* = 0.69 to 0.91), it was decided to retain only the natural logarithm of per capita GDP for 2012–2014 (*M* = 10.44, *SD* = 0.22) in order to reduce issues related to multicollinearity. This variable was provided by the German Federal Office of Statistics (2020), and is a measure of the economic performance of federal states, with higher levels indicating stronger economic performance.

### Analyses

#### Approach for Analyzing a Group-Level Outcome in a Multilevel Model

We are interested in the relationship between individual levels of intergroup anxiety, empathy, and political orientation and federal state levels of acceptance of diversity, while also accounting for the structural properties of the federal states individuals live in. While multilevel models regressing individual-level outcomes on both individual- and group-level predictors (i.e., macro-micro models) have become quite standard in the social sciences for analyzing processes that occur in hierarchical systems (e.g., individuals nested within groups), there is much less of a consensus on the best analysis method for regressing group-level outcomes on individual-level predictors (i.e., micro-macro models; Becker et al., 2018). In the past, a typical approach involved aggregating the individual-level predictors to the group mean, and then using these aggregates as predictors for the group-level outcome. However, this has been criticized in the literature for leading to biased estimates and increasing the risk of invalid conclusions (Snijders and Bosker, 1999; Croon and van Veldhoven, 2007).

We therefore choose to follow the two-step approach outlined by Becker et al. (2018) for analyzing group-level outcomes in multilevel models.^3^ The first step is a standard multilevel analysis regressing the micro-level predictor of interest (i.e., intergroup anxiety, empathy, or political orientation) on all other micro- and macro-level covariates. In the second step, the group-level residuals from the first step are used as a predictor in the group-level linear regression of the group-level outcome (i.e., acceptance of diversity). The effects of these residuals on acceptance of diversity can be interpreted as the aggregated effect of the micro-level predictor, net of all individual- and group-level covariates. Not only does this approach provide a better estimate than the group mean aggregate (since group-level variance is already net of individual-level variance), it also considers both: (1) the mechanisms linking the structural characteristics of the federal states to individuals’ intergroup anxiety, empathy, or political orientation; and (2) the mechanism transforming individuals’ intergroup anxiety, empathy, or political orientation into the creation of the federal state characteristic of acceptance of diversity (Becker et al., 2018).

#### Methods of Analysis

In step one, we fit a series of three multilevel linear regression models each for intergroup anxiety, empathy, and political orientation using the *mixed* command of Stata 16 (StataCorp, 2019) and applying the weights described in “The Data Set” to the individual level data.^4^ The null models (M0_1_) were empty and separated the group-level residuals of intergroup anxiety, empathy, or political orientation from the individual-level residuals. The next models (M1_1_) added the macro-level covariate of per capita GDP, and the final models (M2_1_) added all remaining micro-level predictors and covariates (age, education, etc.).

In step two, we fit another series of three group-level linear regressions for each micro-level predictor of interest with Stata 16’s *regress* command. The first models (M1_2_) predict acceptance of diversity at the federal state level using the group-level residuals from the multilevel null model described above for intergroup anxiety, empathy, or political orientation. The next models (M2_2_) then add the group-level residuals accounting for the macro-level covariate of per capita GDP, and the final models (M3_2_) include the group-level residuals accounting for all macro-covariates as well as remaining micro-level predictors and covariates.

## Results

### Step One: Linking Structural Characteristics to Individual Perceptions

In step one, the null models (M0_1_) for intergroup anxiety, empathy, and political orientation are intercepts-only models (see Table 3 for complete results), providing information on the percentage of total variation in these variables that have to do with the federal state context (i.e., the intra-class correlation *ρ*). The assumption that federal states demonstrate similarities on these variables decreases as the intra-class correlation increases. Our analysis finds minimal context effects for each of the individual-level predictors: ranging from empathy (*ρ* < 0.01) to intergroup anxiety (*ρ* = 0.02) and political orientation (*ρ* = 0.02). In sum, 2% of the variation in intergroup anxiety or political orientation originate from differences at the federal state level, and even less of the variation in empathy is due to those differences.

**Table 3:** Step one: multilevel empty model regressions of intergroup anxiety, empathy, and political orientation: Model 0_1_.

Predictor	IGA	Empathy	PO
	*b*(*se*)	*b*(*se*)	*b*(*se*)
***Level: Federal state (*N_2_* = 16)***			
Intercept	4.33^***^ (0.07)	4.15^***^ (0.02)	4.38^***^ (0.10)
Federal state variance	0.05	0.00	0.11
Individual variance	2.56	0.43	4.38
AIC	10,780	5,688	12,301

Random intercept model. IGA, intergroup anxiety; and PO, political orientation.

*** *p* < 0.001.

All regression coefficients are unstandardized. Standard errors in parentheses.

In the next model (M1_1_), the group-level covariate of per capita GDP was added, indicating only a significant (negative) effect of per capita GDP on individual levels of intergroup anxiety (see Table 4 for complete results). In the final model (M2_1_), the remaining individual-level predictors and sociodemographic covariates were added to the analysis. Accounting for these does not change the relationship between per capita GDP and intergroup anxiety, empathy, or political orientation, but does indicate better model fit (see Table 5 for complete results). In sum, higher per capita GDP at the federal state level is negatively related to individual intergroup anxiety, but it is not related to individual empathy or political orientation.

**Table 4:** Step one: multilevel regressions of intergroup anxiety, empathy, and political orientation on macro-level covariate: Model 1_1_.

Predictor	IGA	Empathy	PO
	*b*(*se*)	*b*(*se*)	*b*(*se*)
***Level: Federal state (N_2_ = 16)***			
Intercept	11.35^***^ (2.24)	3.36^***^ (0.81)	5.68 (4.32)
(ln)per capita GDP	−0.67^**^ (0.21)	0.08 (0.08)	−0.12 (0.42)
Federal state variance	0.02	0.00	0.11
Individual variance	2.57	0.43	4.38
AIC	10,774	5,689	12,302

Random intercept model. IGA, intergroup anxiety; PO, political orientation; and AIC, Akaike information criterion.

** *p* < 0.01.

*** *p* < 0.001.

All regression coefficients are unstandardized. Standard errors in parentheses.

**Table 5:** Step one: multilevel regressions of intergroup anxiety, empathy, and political orientation on macro- and micro-level covariates: Model 2_1_.

Predictor	IGA	Empathy	PO
	*b*(*se*)	*b*(*se*)	*b*(*se*)
***Level: Federal state* (*N_2_* = *16)***			
Intercept	10.31^***^ (2.53)	3.33^***^ (0.78)	4.91 (4.15)
(ln)per capita GDP	−0.59^*^ (0.24)	0.07 (0.07)	−0.05 (0.41)
***Level: Individual (N_1_ = 2,869)***			
Intergroup anxiety		−0.04^***^ (0.01)	0.31^***^ (0.03)
Empathy	−0.22^***^ (0.04)		−0.35^***^ (0.08)
Political orientation	0.18^***^ (0.02)	−0.03^***^ (0.01)	
Age (years)	0.06^***^ (0.01)	0.02^*^ (0.00)	−0.03 (0.02)
Age (squared)	−0.00^***^ (0.00)	−0.00 (0.00)	0.00 (0.00)
Female	0.14 (0.08)	0.23^***^ (0.03)	−0.15 (0.17)
Marital status: married or cohabitating	Ref.	Ref.	Ref.
Marital status: divorced, separated or widowed	−0.00 (0.09)	−0.08^*^ (0.04)	−0.28^*^ (0.12)
Marital status: single	−0.08 (0.12)	−0.04 (0.05)	−0.37^*^ (0.15)
Education: University degree	Ref.	Ref.	Ref.
Education: vocational training	0.52^***^ (0.06)	−0.02 (0.03)	0.18 (0.12)
Education: no completed secondary education	0.35^***^ (0.09)	−0.04 (0.07)	0.06 (0.16)
Employed	−0.31^***^ (0.09)	0.02 (0.03)	0.16 (0.15)
Migration background	0.20^**^ (0.07)	0.07 (0.04)	−0.08 (0.10)
Federal state variance	0.03	0.00	0.09
Individual variance	2.26	0.40	3.99
AIC	10,437	5,497	12,060

Random intercept model. IGA, intergroup anxiety; PO, political orientation; AIC, Akaike information criterion; and Ref., Reference group.

* *p* < 0.05.

** *p* < 0.01.

*** *p* < 0.001.

All regression coefficients are unstandardized. Standard errors in parentheses.

### Step Two: Transforming Individuals’ Perceptions to Structural Characteristics

In step two, the first models (M1_2_) predict acceptance of diversity using the group-level residuals from the multilevel null models. This accounts for the variance of the individual levels of intergroup anxiety, empathy, and political orientation, leaving only a negative significant relationship between intergroup anxiety and acceptance of diversity (see Table 6 for complete results). This negative significant relationship remains between intergroup anxiety and acceptance of diversity even after including the residuals correcting for group-level covariates (M2_2_) as well as the remaining individual-level predictors and covariates (M3_2_; see Tables 7, 8 for complete results). In summary, higher levels of intergroup anxiety are related to lower levels of federal state acceptance of diversity, even after accounting for structural characteristics of the federal states and individual sociodemographic characteristics. No relationships were found between empathy or political orientation and acceptance of diversity. These findings confirm Hypothesis 1, but do not confirm Hypotheses 2 or 3.

**Table 6:** Step two: linear regression of acceptance of diversity on residuals of null model (M0_1_) on intergroup anxiety, empathy, and political orientation: Model 1_2_.

Predictor	IGA	Empathy	PO
	*b*(*se*)	*b*(*se*)	*b*(*se*)
Intercept	67.29^***^ (0.48)	67.29^***^ (0.91)	67.29^***^ (0.88)
Residuals (null model, M0_1_)	−15.72^***^ (2.47)	3.42 (24.76)	−3.15 (3.10)
AIC	72.54	92.69	91.71
BIC	74.86	95.01	94.03

IGA, intergroup anxiety; PO, political orientation; AIC, Akaike information criterion; and BIC, Bayesian information criterion.

*** *p* < 0.001.

All regression coefficients are unstandardized. Standard errors in parentheses.

**Table 7:** Step two: linear regression of acceptance of diversity on residuals of M1_1_ on intergroup anxiety, empathy, and political orientation: Model 2_2_.

Predictor	IGA	Empathy	PO
	*b*(*se*)	*b*(*se*)	*b*(*se*)
Intercept	67.29^***^ (0.78)	67.29^***^ (0.90)	67.29^***^ (0.89)
Residuals (Model 1_1_)	−16.70^*^ (6.85)	−13.37 (26.24)	−2.41 (3.14)
AIC	87.66	92.46	92.14
BIC	89.97	94.78	94.45

IGA, intergroup anxiety; PO, political orientation; AIC, Akaike information criterion; and BIC, Bayesian information criterion.

* *p* < 0.05.

*** *p* < 0.001.

All regression coefficients are unstandardized. Standard errors in parentheses.

**Table 8:** Step two: linear regression of acceptance of diversity on residuals of M1_2_ on intergroup anxiety, empathy, and political orientation: Model 3_2_.

Predictor	IGA	Empathy	PO
	*b*(*se*)	*b*(*se*)	*b*(*se*)
Intercept	67.29^***^ (0.80)	67.29^***^ (0.90)	67.29^***^ (0.89)
Residuals (Model 1_2_)	−12.88^*^ (5.96)	−12.94 (27.14)	−2.60 (3.34)
AIC	88.62	92.49	92.12
BIC	90.94	94.80	94.44

IGA, intergroup anxiety; PO, political orientation; AIC, Akaike information criterion; and BIC, Bayesian information criterion.

* *p* < 0.05.

*** *p* < 0.001.

All regression coefficients are unstandardized. Standard errors in parentheses.

## Discussion

Growing diversity has become a reality in the majority of Western societies since World War II. Research interested in its impact on the individual as well as on the society mostly focuses on ethnic, cultural, and religious variables. However, this narrow conception does not reflect the actual breadth of diversity in most communities. While many see increasing diversity as a chance for growth, development, and innovation, others are worried about the erosion of social cohesion and the loss of defining elements of their society, such as established customs and social rules. It is indicative therefore to go beyond ethnic origin and religion when we speak about diversity in the proverbial WEIRD societies, and include other relevant aspects. Only if we take this multidimensionality into account, is it possible to understand the mechanisms that foster or hamper the acceptance of diversity in a given community. Do individual factors play a role alongside structural variables? By building a bridge between psychological and sociological approaches, we sought to answer the question of whether *acceptance of diversity in social entities*, our target variable, is related to prevailing mentalities once their social structure is accounted for.

In designing our study, we took into account some of the shortcomings of prior research. First, instead of operationalizing diversity only in ethnic or cultural-religious terms, we used a multidimensional conceptualization to measure acceptance of diversity in a broader understanding, including the following seven dimensions: age differences, disability, gender, sexual orientation and gender identity, ethnicity, religion, and economic disadvantage (c.f. Arant et al., 2019). Second, although unusual for a psychological endeavor, instead of focusing only on individual factors impacting the acceptance of diversity, we also took the societal level into account. By investigating the role of individual and societal factors, we attempted to reflect the living conditions of individuals in communities more comprehensively to increase the understanding of what influences the acceptance of diversity at the group level. Third, psychological research often has to rely on relatively small and/or haphazard samples. To draw more accurate conclusions about the general population, the data for our analyses stem from a large, representative survey in Germany.

How do the results of the present study contribute to understanding which factors play a vital role in the acceptance of diversity today? Firstly, as hypothesized, we found that when study participants express higher levels of intergroup anxiety, their German federal states of residence exhibit lower levels of diversity acceptance. This fits with prior psychological research (Stephan and Stephan, 1985; Laurence et al., 2019). Fear of others or “the unknown” leads to various problematic cognitive, emotional and behavioral outcomes, such as negative attitudes, stereotypes, and beliefs, fear, threat, or hate as well as avoidance or aggression toward the outgroup at the individual level (Stephan, 2014) and according to our results, spills over, so-to-speak, into the level of sociopolitical entities. Our study, thus, confirms the well-established link between negative emotions toward outgroups and low levels of diversity acceptance. Second, our analyses showed that the economic situation in the community (measured as per capita GDP) functions as a catalyst for intergroup anxiety. Broader economic issues intensify the fear of others, a link that also has been found before (Oliver and Mendelberg, 2000; Oliver and Wong, 2003; Laurence, 2011; Laurence et al., 2019).

Finally, we obtained two unexpected results. Contrary to our hypothesis, we could not confirm the expected positive effect of individual empathy on acceptance of diversity on the aggregate level. At first glance, this result is puzzling since research consistently shows that empathy does have a positive effect on individual attitudes and behavior toward others (Vezzali et al., 2010; Butrus and Witenberg, 2013). However, readers should note that we are not predicting individual-level acceptance of diversity on the basis of empathy, but community-level (here: state-level) acceptance of diversity on the grounds of levels of empathy among people living in a given federal state. It may be the case that empathy affects the individual level of acceptance of diversity, but does not have a “political” consequence by also affecting “public” levels of diversity acceptance.

The second unexpected finding refers to the political orientation of our study participants. Although previous research is scarce and provides inconclusive results, studies – particularly for the German context – have indicated that right-leaning political orientations go hand-in-hand with higher intergroup anxiety (Rippl et al., 2007; Zeigler-Hill et al., 2020), suggesting that both might be predictors of community-level acceptance of diversity. However, our study could not confirm this. In fact, we found both variables to be completely unrelated when looking at the predictive power of individual political orientations for community-level diversity acceptance, which was close to nil, whereas individual-level intergroup anxiety proved to affect acceptance of diversity on the federal state level to a non-neligible degree. Taken together, neither individual empathy nor political orientation seem to play a vital role for diversity acceptance at the level of the 16 German federal states.

The results of our analyses make an important contribution to the literature and help to frame the work of practitioners on a community level. First and foremost, our study underscores the central role intergroup anxiety plays for the acceptance of diversity. Although this finding is generally not new, investigating this link from a mixed individual-structural perspective puts another emphasis on the relevance anxiety plays in intergroup relations as well as on its strongest antidote: intergroup contact. Research consistently shows that contact helps to lower perceived intergroup threat (Tausch et al., 2007; Pettigrew and Tropp, 2008). Recently, Stephan (2014) summarized the relevant conditions and outcomes: “Specifically, neutral and positive contact should reduce intergroup anxiety because it provides information about outgroups, increases understanding of outgroups, personalizes outgroup members, undermines perceived threats, reduces concerns about rejection or negative behaviors by outgroups, promotes empathy, undercuts negative attitudes and stereotypes, and allows people to develop skills in interacting with outgroup members. Optimally, contact should be long term, involve multiple outgroup members, and occur in different social contexts” (p. 11).

However, promoting intergroup contact is a challenge in many communities because resources – both monetary and in terms of personnel – are usually scarce. As our analyses show, this is particularly problematic for communities that are already disadvantaged, since lower economic status of a community increases intergroup anxiety and therefore exacerbates the problematic link between intergroup anxiety and diversity acceptance. At the same time, empathy training, as for example offered by Roots of Empathy, an originally Canadian civil society organization (Gordon, 2009), is likely to engage in a steep uphill struggle with its training programs for children. Our study offers no evidence that there is a spill-over effect from individual levels of empathy onto the acceptance of diversity in a community. What fosters optimism is, however, that acceptance of diversity in a community is broadly unaffected by the political orientation of its residents. Therefore, at least, the political stance does not bar the way to an accepting society. The results of our study underline the pressing necessity of governments to invest in measures that help increasing neutral and positive intergroup contact, particularly in socioeconomically weaker communities if they want to secure the social cohesion of their societies.

### Limitations

Besides its conceptual and methodological strength, the present study comes with various limitations. First, in being a case study of Germany its generalizability is limited, particularly since previous research has found the German context to differ from other European settings or the United States (Rippl et al., 2007; Stolle et al., 2013). Furthermore, although analyses were carried out on the basis of a large, population representative sample, which is still exceptional for psychological research endeavors, the available data only allowed for a limited multilevel analysis. To compare different sociopolitical entities within Germany with enough statistical power, we had to choose rather large units, namely the 16 federal states. However, a much finer subdivision into regions, municipalities, or even neighborhoods might have detected relevant variations in empathy or political orientation that remained uncovered in our analysis because their effects on acceptance of diversity were canceled out due to the chosen aggregation level. Another shortcoming that affects much similar research is the question of change. The perception of diversity constantly changes in a society, not only because of actual increases or decreases in diversity, but also due to the perceptions of the members of a society. How those changes impact the acceptance of diversity can only be investigated with longitudinal/panel data. Therefore, much larger, longitudinal datasets are indispensable for the understanding of the interplay between societal and individual processes for the acceptance of diversity.

### Conclusion

Ensuring and improving social cohesion have become important goals in public discourse, in political decision-making, and also in academic work. In the search for relevant factors that influence social cohesion, diversity has crystallized to be one of the central variables both on a structural as well as on an individual level. Since increasing diversity is a fact in Western societies, this paper argued that identifying factors that contribute to high vs. low levels of subjective acceptance of diversity is much more fruitful than focusing on the objective level of diversity in social entities. By taking a multilevel approach, our study showed that individuals’ intergroup anxiety is the key to diversity acceptance in the community. Our approach also takes into account the level of economic prosperity in the community. Surprisingly, neither individual levels of empathy nor political orientation played a role. Although at first sight, the partial “non-findings” of our study may seem disappointing, our research carries a clear message that cannot be emphasized enough: fear is the biggest enemy of the acceptance of others. Whereas empathy and political orientation are often seen as rather stable personality attributes that are difficult to change, there is a well-proven remedy for intergroup anxiety: “Intergroup *contact*, and not merely living together, apart is crucial to this end.” (Hewstone, 2015, p. 434, emphasis in the original). Creating communities where people meet each other and interact on a neutral or positive ground is the best precondition for a future in which diversity with all its facets is accepted, which in turn helps to ensure or build strong and cohesive societies. Therefore, our study underscores that aside from structural properties of social entities, individual mentalities do matter in determining how strong social cohesion is in a society and how it can prosper.

## Data Availability Statement

The multilevel dataset used for the analysis, as well as the data dictionary are accessible for the scientific community through the Open Science Framework and can be downloaded using following link: https://osf.io/ryfxs/ or doi: 10.17605/OSF.IO/RYFXS.

## Ethics Statement

Ethical review and approval was not required for the study on human participants in accordance with the local legislation and institutional requirements. Written informed consent from the participants’ legal guardian/next of kin was not required to participate in this study in accordance with the national legislation and the institutional requirements.

## Author Contributions

KB, RA, and ML contributed to the conception and design of the study. RA and ML conducted the literature search and wrote the first draft of the manuscript. ML performed the statistical analysis. KB contributed with his expertise in the research field. RA and KB designed the data collection instruments. RA coordinated and supervised data collection. All authors contributed to the article and approved the submitted version.

### Conflict of Interest

The authors declare that the research was conducted in the absence of any commercial or financial relationships that could be construed as a potential conflict of interest.

## Footnotes

^1^Example items for the seven dimensions of the BDB can be found in Table 2.

^2^Cronbach’s alpha of internal consistency ranged from 0.37 for “disability” with three items, to 0.79 for “sexual orientation and gender identity” with four items. By making use of the application of the Spearman-Brown formula (pp. 223–226) of Nunnally (1967), a relative threshold for Cronbach’s alpha can be attained which accounts for the number of items. In this case, an alpha of 0.10 times, the number of items is proposed as a sufficient threshold (e.g., Kotzur et al., 2017). Applying this relative threshold, all of the constructed dimensions of acceptance of diversity indicated sufficient quality.

^3^The multilevel structural equation modeling approach, which replaces manifest with latent aggregation for micro-level predictors, is another possible approach. However, due to the small number of groups in our analysis (*N_2_* = 16), it would not have been recommended due to the biased estimates of the macro-level standard errors (Hox, 2010).

^4^No weights were applied at the group level because all federal states were included in the data collection and no sampling of federal states was necessary (e.g., Carle, 2009).
