# Corrigendum: Acceptance of Diversity as a Building Block of Social Cohesion: Individual and Structural Determinants

**DOI:** 10.3389/fpsyg.2021.685063

**Published:** 2021-04-22

**Authors:** Regina Arant, Mandi Larsen, Klaus Boehnke

**Affiliations:** ^1^Department of Psychology and Methods, Jacobs University Bremen, Bremen, Germany; ^2^Bremen International Graduate School of Social Sciences (BIGSSS), Jacobs University Bremen, Bremen, Germany; ^3^Center for Sociocultural Research, National Research University – Higher School of Economics, Moscow, Russia

**Keywords:** acceptance of diversity, intergroup anxiety, empathy, political orientation, social cohesion, group-level outcome, multilevel analysis

There was an important omission in the Acknowledgments section as published. There must be a reference to an institutional funder of the work on the article. The corrected Acknowledgments section is shown below.

**ACKNOWLEDGMENTS**

The article was prepared within the framework of the HSE University Basic Research Program. We thank Caroline Schnelle for her tireless assistance in digging up the pertinent social science literature, and Dr. Georgi Dragolov for his invaluable help with data handling.

The authors apologize for this error and state that this does not change the scientific conclusions of the article in any way. The original article has been updated.

